# Astrocyte-mediated inflammatory responses in traumatic brain injury: mechanisms and potential interventions

**DOI:** 10.3389/fimmu.2025.1584577

**Published:** 2025-05-08

**Authors:** Haifeng Zhang, Xian Zhang, Yan Chai, Yuhua Wang, Jianning Zhang, Xin Chen

**Affiliations:** ^1^ Department of Neurosurgery, Tianjin Medical University General Hospital, Tianjin, China; ^2^ Tianjin Neurological Institute, Key Laboratory of Post-Trauma Neuro-Repair and Regeneration in Central Nervous System, Ministry of Education, Tianjin, China

**Keywords:** traumatic brain injury, astrocyte, inflammasome, pro-inflammatory cytokines, chemokines, therapy

## Abstract

Astrocytes play a pivotal role in the inflammatory response triggered by traumatic brain injury (TBI). They are not only involved in the initial inflammatory response following injury but also significantly contribute to Astrocyte activation and inflammasome release are key processes in the pathophysiology of TBI, significantly affecting the progression of secondary injury and long-term outcomes. This comprehensive review explores the complex triggering mechanisms of astrocyte activation following TBI, the intricate pathways controlling the release of inflammasomes from activated astrocytes, and the subsequent neuroinflammatory cascade and its multifaceted roles after injury. The exploration of these processes not only deepens our understanding of the neuroinflammatory cascade but also highlights the potential of astrocytes as critical therapeutic targets for TBI interventions. We then evaluate cutting-edge research aimed at targeted therapeutic approaches to modulate pro-inflammatory astrocytes and discuss emerging pharmacological interventions and their efficacy in preclinical models. Given that there has yet to be a relevant review elucidating the specific intracellular mechanisms targeting astrocyte release of inflammatory substances, this review aims to provide a nuanced understanding of astrocyte-mediated neuroinflammation in TBI and elucidate promising avenues for therapeutic interventions that could fundamentally change TBI management and improve patient outcomes. The development of secondary brain injury and long-term neurological sequelae. By releasing a variety of cytokines and chemokines, astrocytes regulate neuroinflammation, thereby influencing the survival and function of surrounding cells. In recent years, researchers have concentrated their efforts on elucidating the signaling crosstalk between astrocytes and other cells under various conditions, while exploring potential therapeutic interventions targeting these cells. This paper highlights the specific mechanisms by which astrocytes produce inflammatory mediators during the acute phase post-TBI, including their roles in inflammatory signaling, blood-brain barrier integrity, and neuronal protection. Additionally, we discuss current preclinical and clinical intervention strategies targeting astrocytes and their potential to mitigate neurological damage and enhance recovery following TBI. Finally, we explore the feasibility of pharmacologically assessing astrocyte activity post-TBI as a biomarker for predicting acute-phase neuroinflammatory changes.

## 1 Introduction

Traumatic brain injury (TBI) is a major global health burden and a leading cause of death and long-term disability across all populations (1). TBI is characterized by an initial mechanical insult to the brain parenchyma, which subsequently initiates intricate secondary injury mechanisms. These secondary processes can persist and progress over extended temporal scales, spanning from hours to years post-injury. Within the spectrum of secondary factors, the inflammatory response emerges as a critical determinant of tissue fate and long-term neurological sequelae (2–4). This inflammatory cascade response exhibits a biphasic nature, initially serving a neuroprotective function before rapidly transitioning into a potentially deleterious process (5–7). In mouse models, the acute phase of traumatic brain injury (TBI) typically refers to the early stage occurring within hours to days post-injury, particularly during the critical 24–72 hour window (8, 9). This phase is characterized by rapid progression of pathological damage, including direct mechanical trauma-induced neuronal structural destruction, tissue edema, and rapid initiation of inflammatory responses (9). Notably, plasma levels of the astrocytic marker GFAP and axonal injury marker NFL show significant elevation (8).The acute phase response of TBI exhibits specific characteristics distinguishable from other neurological disorders: First, TBI’s acute response has a defined temporal profile (24–72 hours), contrasting sharply with the chronic inflammatory processes (lasting months to years) observed in neurodegenerative diseases such as Alzheimer’s and Parkinson’s disease (8). Second, TBI-induced elevations in GFAP and NFL during the acute phase are characteristic, whereas these markers may show delayed or insignificant changes in other conditions (1). Furthermore, TBI-specific pathological processes include blood-brain barrier (BBB) disruption, local ischemia, and mechanical cell necrosis, features that are rare or manifest through different mechanisms in other neurological conditions (10, 11). During the acute phase, astrocytes rapidly enter an activated state, characterized by cellular hypertrophy, proliferation, and significant upregulation of GFAP expression (12). Activated astrocytes simultaneously secrete pro-inflammatory factors such as IL-6, TNF-α, and MMP9, further exacerbating BBB damage and neuronal oxidative stress (13). This process is primarily mediated through the activation of TLR2/p44/42 MAPK signaling and NF-κB pathways (14). While excessive inflammatory response may lead to neuronal death and tissue edema, the formation of glial scarring effectively isolates the injured area, limiting inflammatory spread (12, 15). In the subacute phase (3 days to several weeks), levels of pro-inflammatory factors (TNF-α, IL-6) gradually decrease, while anti-inflammatory factors (IL-10) and neuroprotective molecules (IGF-1) show increased expression (16). Astrocytes begin secreting chondroitin sulfate proteoglycans (CSPGs) and laminins, which both inhibit inflammatory spread and promote angiogenesis (17). Through STAT3 and TGF-β signaling pathway regulation, these processes promote synaptic remodeling, neurogenesis, and axonal regeneration, while glial scarring serves dual roles in suppressing inflammation and facilitating repair (17). Notably, the JAK/STAT signaling pathway gradually transitions from promoting inflammation to regulating repair-related gene expression (18).In the chronic phase (weeks to months), some astrocytes continue to express pro-inflammatory markers such as C3d, maintaining chronic neuroinflammation (19). Simultaneously, damaged neurons activate astrocytes through the DAMPs-RIPK3 signaling pathway, leading to persistent inflammation and neurodegeneration (20). During this phase, anti-inflammatory astrocyte subpopulations promote axonal regeneration through S100A10 protein secretion, although excessive glial proliferation may inhibit neural plasticity, ultimately resulting in cognitive dysfunction (21). Epigenetic regulatory mechanisms (including DNA methylation and histone modifications) significantly influence astrocyte phenotype, determining their pro- or anti-inflammatory fate (22). Additionally, abnormal opening of Cx43 hemichannels further exacerbates chronic inflammation, a phenomenon closely associated with neurological conditions such as multiple sclerosis (23).

Building on the previous discussion, we now turn our attention to a key cellular component of the neuroinflammatory cascade response following TBI: astrocytes. Historically regarded as supportive cells in central nervous system (CNS) function, astrocytes have now emerged as pivotal regulators of the brain’s innate immune response. Recent advancements in neuroscience have elucidated the multifaceted changes of astrocytes in both physiological and pathological states (24). Astrocytes, exhibit remarkable heterogeneity, with their morphology and functions dynamically changing across brain regions, developmental stages, and pathological conditions. In response to injury or inflammation, these cells transform into a reactive state (reactive astrogliosis) and differentiate into distinct subtypes, most notably the neurotoxic A1 phenotype and the neuroprotective A2 phenotype. A1 astrocytes, first reported by Liddelow et al., are primarily induced by activated microglia through the secretion of inflammatory mediators, including IL-1α, TNF-α, and C1q. These factors subsequently activate cells through NF-κB and JAK/STAT signaling pathways (25). A1 astrocytes are characterized by significant upregulation of complement component C3, MX1, and pro-inflammatory cytokines (such as IL-6 and TNF-α) (26). Notably, these cells lose their supportive functions and instead secrete neurotoxic substances, leading to the death of neurons and oligodendrocytes (27). In contrast, A2 astrocytes are predominantly induced under ischemic or traumatic conditions, with their formation mechanism involving STAT3 activation and ROS-mediated NF-κB inhibition (28, 29). These cells are molecularly characterized by high expression of S100A10, EMP1, and anti-inflammatory factors (such as Arginase 1 and Nrf2) (29, 30). They promote synaptic formation, neuronal survival, and antioxidant effects through the secretion of neurotrophic factors, including BDNF, VEGF, and bFGF (27). In response to TBI, astrocytes undergo a rapid and profound phenotypic transformation, a process termed reactive astrogliosis (31). This reactive state is characterized by hypertrophy of astrocytic processes, upregulation of intermediate filament proteins (particularly glial fibrillary acidic protein, GFAP), and substantial alterations in gene expression profiles (32, 33). Reactive astrocytes undergo a remarkable functional shift from homeostatic maintenance to active participation in the inflammatory response. This functional plasticity is evidenced by their capacity to synthesize and release a diverse array of pro-inflammatory mediators, including cytokines chemokines, and other inflammatory modulators such as prostaglandins and reactive oxygen species (34–39). Furthermore, astrocytes upregulate the expression of pattern recognition receptors (PRRs), including Toll-like receptors (TLRs) and NOD-like receptors (NLRs), thereby enhancing their responsiveness to damage-associated molecular patterns (DAMPs) released during TBI (40–42). Through the release of chemokines and cytokines, reactive astrocytes promote the recruitment and activation of peripheral immune cells, thus amplifying the inflammatory cascade. In addition, matrix metalloproteinases (MMPs) released by astrocytes exacerbate blood-brain barrier disruption, further promoting neuroinflammation (43). However, the roles of astrocytes in TBI are not uniformly deleterious. These cells also exhibit neuroprotective functions, including scavenging of free radicals and production of neurotrophic factors (44, 45). This duality underscores the complexity and context-dependent nature of the astrocyte response to TBI, emphasizing the necessity for a nuanced approach in targeting astrocyte-mediated inflammation. Recent studies have begun to elucidate the molecular mechanisms governing astrocyte responsiveness in TBI, identifying key signaling pathways such as signal transducer and activator of transcription 3 (STAT3), nuclear factor kappa b (NF-κB), and mitogen-activated protein kinase (MAPK) cascades as potential therapeutic targets (46–48). Moreover, the evolving understanding of astrocyte heterogeneity in health and disease has prompted investigations into specific astrocyte responses to TBI, unveiling novel avenues for targeted interventions.

This review focuses specifically on the substances released by astrocytes following TBI activation and their role in the initiation and propagation of inflammatory cascades. A comprehensive understanding of these mechanisms will elucidate the complex interactions between astrocytes and other components of the neurovascular unit in the context of TBI, illuminate the temporal dynamics of the inflammatory response, and potentially identify critical windows for therapeutic intervention.

## 2 Triggers of astrocyte activation in TBI

**Figure 1:**
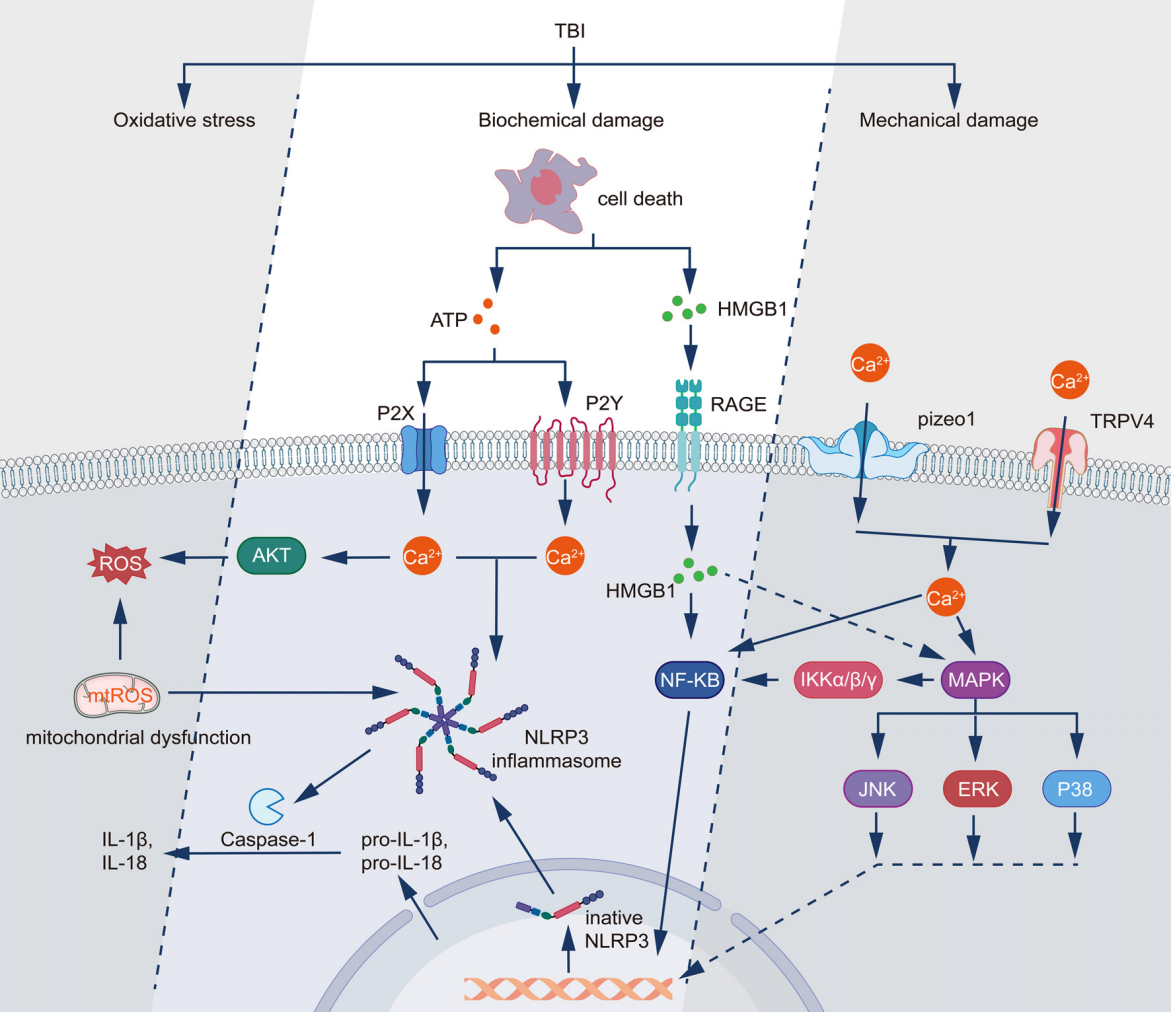
Molecular mechanisms underlying TBI-induced inflammatory responses and cellular damage. TBI triggers three primary pathological processes: mechanical damage, biochemical damage, and oxidative stress. Following mechanical injury, TRPV4 and Piezo1 channels mediate Ca2+ influx, while cell death releases DAMPs including HMGB1 and ATP. HMGB1 activates the RAGE receptor pathway, leading to NF-κB activation through IKKα/β/γ signaling, while simultaneously triggering MAPK cascades (JNK, ERK, and P38). ATP signaling operates through both P2Y and P2X receptors, inducing calcium influx and subsequent activation of AKT and ROS production. The increased intracellular Ca2+ and mitochondrial dysfunction generate mitochondrial ROS (mtROS), which activates the NLRP3 inflammasome complex. This activation leads to caspase-1-mediated processing of pro-IL-1β and pro-IL-18 into their mature forms (IL-1β and IL-18). The interconnected signaling networks culminate in the production of inflammatory mediators and oxidative stress, establishing a feedback loop that potentially exacerbates the initial injury.

Astrocyte activation in the acute phase following TBI is a complex process initiated by multiple interrelated mechanisms. The primary triggers and their associated activation pathways can be broadly categorized into mechanical, biochemical, and cellular responses to injury. Mechanical stimuli initiate the process of acute astrocyte activation. The direct physical force of trauma induces cellular deformation and mechanoreceptor activation in astrocytes. This mechanical stress facilitates the opening of mechanosensitive ion channels, particularly stretch-activated calcium channels, culminating in an influx of calcium ions (49, 50). Elevated intracellular calcium concentration functions as a crucial second messenger, activating a diverse array of signaling cascades and promoting astrocyte reactivity (**Figure 1**) (51). Concomitantly, mechanical disruption of the BBB contributes to astrocyte activation. A compromised BBB permits the infiltration of blood-derived molecules and cells into the brain parenchyma. The primary mechanism by which plasma proteins, such as thrombin and fibrinogen, activate astrocytes is through the interaction of protease-activated receptors (PARs), specifically PAR-1, PAR-3, and PAR-4, with astrocyte surface receptors, triggering activation and proliferation (52). Fibrinogen activates astrocytes through the synergistic action of at least three distinct fibrinolytic proteinase receptors, leading to the expression of proinflammatory cytokines (53). Additionally, the extravasation of immune cells, particularly neutrophils and monocytes, further amplifies the astrocyte activation process through the release of proinflammatory cytokines and chemokines (54).

Biochemical triggers play a crucial role in astrocyte activation following the initial mechanical injury (**Figure 1** Molecular mechanisms underlying TBI-induced inflammatory responses and cellular damage.). The rapid disruption of cellular homeostasis leads to the release of various signaling molecules and ions from damaged cells. Notably, the extracellular concentration of adenosine triphosphate (ATP) increases substantially following TBI due to cell damage and lysis. Astrocytes express a variety of purinergic receptors, including P2XR and P2YR, which are activated by extracellular ATP (55, 56). Stimulation of these receptors induces calcium influx and initiates a signaling cascade that promotes astrocyte responsiveness (57). Concurrently, ATP released by astrocytes modulates synaptic transmission. ATP activates P2XR in neighboring neurons, thereby enhancing excitatory signaling (58). Further studies have revealed that ATP induces graded activation responses in astrocytes, including cell proliferation, stellate morphogenesis, and shape remodeling (59). Experimental evidence demonstrates that the increase in ATP persists for 24 hours post-brain cell death, suggesting a sustained release from necrotic post-inflammatory tissues (56). Oxidative stress, a hallmark of acute TBI, represents another potent biochemical trigger for astrocyte activation. Reactive oxygen species (ROS) and reactive nitrogen species (RNS) are produced as a result of mitochondrial dysfunction, NADPH oxidase activation, and inflammatory responses (60). Astrocytes possess redox-sensitive transcription factors, such as nuclear factor erythroid 2-related factor 2 (Nrf2) and NF-κB, which respond to oxidative stress by inducing the expression of genes associated with astrocyte activation and neuroinflammation. Notably, activation of Nrf2 confers neuroprotection against oxidative stress-induced injury, whereas activation of NF-κB elicits a series of proinflammatory responses that culminate in neuronal death (61, 62). Cellular debris and DAMPs released by injured or necrotic cells serve as potent triggers of astrocyte activation. These molecules, including high mobility group box 1 (HMGB1), ATP, and heat shock proteins, bind to PRRs on astrocytes, such as the Toll-like receptor (TLR) and the receptor for advanced glycation end-products (RAGE) (63). The activation of these receptors initiates a signaling cascade involving MAPK and NF-κB, leading to the production of proinflammatory cytokines and chemokines, thereby perpetuating the process of astrocyte activation (summarized in **Figure 1**).

The acute phase response to TBI involves the rapid production and release of cytokines and growth factors from diverse cell types, including neurons, microglia, and endothelial cells. These factors, including substance P, interleukin-1β (IL-1β), tumor necrosis factor-α (TNF-α), and transforming growth factor-β (TGF-β), bind to their respective receptors on astrocytes, activating downstream signaling pathways that promote astrocyte proliferation, hypertrophy, and adoption of the reactive phenotype (64). These triggers and mechanisms operate within a complex, interconnected network of signaling events rather than in isolation. For instance, mechanical disruption of the BBB facilitates the infiltration of blood-borne molecules and contributes to ROS production and proinflammatory mediator release, which further compromise BBB integrity. Excitotoxicity in astrocytes promotes elevated intracytoplasmic calcium ion levels, enhanced calcium transients, and increased frequency of calcium oscillations (65). The accumulation of calcium ions in mitochondria depletes the mitochondrial membrane potential, resulting in impaired ATP production. Formation of the mitochondrial permeability transition pore (MPTP) facilitates the release of cytotoxic factors, including cytochrome c and ROS, into the cytoplasm, exacerbating oxidative stress and perpetuating the activation cycle (66, 67).

Activated astrocytes themselves become sources of diverse signaling molecules, including cytokines, chemokines, and growth factors. This phenomenon creates feedforward loops that amplify and sustain the activation process. For instance, astrocyte-secreted brain endothelin-1 (ET-1) acts on the astrocyte endothelin B (ETB) receptor, maintaining and enhancing astrocyte activation through the autocrine pathway and by upregulating ETB receptor expression (68). Connective tissue growth factor (CTGF) can be Activated autocrinally and by astrocytes through the ASK1-p38/JNK-NF-κB/AP-1 pathway, promoting astrocyte-mediated inflammatory responses (69). These factors further activate astrocytes and promote the release of additional proinflammatory mediators, perpetuating the inflammatory cascade.

The temporal dynamics of these triggers and mechanisms warrant careful consideration. Certain processes, such as mechanoreceptor activation and glutamate release, occur almost instantaneously following injury, whereas others, including immune cell infiltration and the full manifestation of oxidative stress, develop over a period of hours to days. This temporal heterogeneity contributes significantly to the complexity and progression of astrocyte activation in the acute phase following TBI. These triggers collectively drive astrocytes toward a pro-inflammatory phenotype, subsequently leading to the release of inflammatory mediators through multiple pathways.

## 3 Key substances released by activated astrocytes

Astrocytes play a crucial role in neuroinflammatory responses within the central nervous system (CNS), with their key inflammatory mediators exhibiting distinct release mechanisms, signaling pathways, and functional localization compared to those produced by other immune cells. Astrocytes, activated through IL-1α stimulation or endoplasmic reticulum stress pathways, produce IL-1β, leading to neurotoxic effects (70, 71). In contrast, microglia, as CNS-resident immune cells, generate IL-1β through the NLRP3 inflammasome (72, 73), while endothelial cells participate in IL-1β release during vascular injury (74), and infiltrating monocyte-macrophages activate inflammasomes via TLR signaling following BBB disruption (75). Astrocytes release TNF-α through the NF-κB signaling pathway and act synergistically with COX2 to promote inflammation (71), whereas microglia rapidly secrete TNF-α following glutamate receptor activation, directly triggering neuronal death (72). Infiltrating M1 macrophages exhibit a pro-inflammatory phenotype in the CNS, releasing high levels of TNF-α (76), while neutrophils release TNF-α through NETs (77). In their reactive state, astrocytes release IL-6 via the DAMPs-TLR4 signaling axis (78), while microglial-derived extracellular vesicles (MDEVs) carry IL-6, activating autocrine and paracrine inflammatory cycles (71). Infiltrating M1 macrophages secrete IL-6 at injury sites (76), and neurons also express IL-6 during extended survival periods following TBI (29).Astrocytes trigger CCL2 release through TLR/IL-1R signaling, recruiting monocytes (64, 79), while microglia, serving as primary chemokine sources, facilitate peripheral immune cell migration across the BBB (71, 75). Infiltrating monocytes are recruited to the CNS via the CCL2-CCR2 axis and further secrete CCL2, establishing a positive feedback loop (64, 75). Astrocytes upregulate CXCL10 through JAK2/STAT3 and NF-κB pathways, promoting T cell infiltration (71), while microglia secrete CXCL10 under LCN2 stimulation, participating in cell migration and inflammation amplification (77). CXCR3+ T cells enter the CNS through CXCL10-mediated chemotaxis, enhancing local immune responses (80), and dendritic cells (DCs) may mediate adaptive immune regulation through CXCL10 in late-stage TBI (77). Astrocytes and microglia form inflammatory networks following TBI, exemplified by microglial extracellular vesicles activating astrocytic IL-6 and TNF-α release (71). Infiltrating macrophages share phenotypic overlap (M1/M2) with resident immune cells (such as microglia) but may exhibit complementary or antagonistic functions (76, 81).

### 3.1 Pro-inflammatory cytokines

The mechanisms and pathways underlying the production and release of pro-inflammatory factors by reactive astrocytes are intricate, encompassing diverse molecular cascades and intercellular interactions. Upon activation in response to CNS injury or disease, astrocytes generate and release a diverse array of pro-inflammatory factors, thereby playing a pivotal role in the initiation and progression of neuroinflammation. The key pro-inflammatory factors secreted by reactive astrocytes can be broadly classified into four categories: cytokines, chemokines, complement components, and miscellaneous factors. Elucidating the temporal release patterns and spheres of influence of various pro-inflammatory factors facilitates the precise definition of therapeutic windows and target regions for the treatment of CNS injuries and diseases, potentially enhancing patient outcomes through tailored interventions. The outcomes of these studies are summarized in **Table 1**.

**Table 1:** Inflammatory receptor activation pathways in astrocytes following traumatic brain injury.

Receptor Family	Specific Receptor	TBI-induced Ligands/Activators	Signaling Pathways	Pro-inflammatory Mediators	Impact on Secondary Injury	Cite
Pattern Recognition Receptors	TLR4	HMGB1 (necrotic cells); HSP60/70 (cellular stress); Fibrinogen (BBB disruption); S100β (astrocytic damage);	MyD88-dependent pathway: IRAK1/4, TRAF6, TAK1, NF-κB/MAPK activation; TRIF-dependent pathway: IRF3-mediated response	Pro-inflammatory cytokines: TNF-α, IL-1β, IL-6; Chemokines: CCL2, CXCL10; Inflammatory mediators: NO, PGE2	Exacerbation of neuronal death; BBB dysfunction; Cerebral edema formation; Axonal injury progression	(82–86)
	TLR2	HMGB1; Cellular debris; HSP60/70 (cellular stress)	MyD88-dependent signaling cascade; NF-κB nuclear translocation	Pro-inflammatory cytokines: TNF-α, IL-1β, IL-6; Proteases: MMP-9	Neuroinflammation amplification; BBB integrity compromise; Impaired neuroregeneration	(13, 86–88)
Cytokine Receptors	IL-1R	IL-1α (mechanical trauma); IL-1β (inflammatory response)	MyD88 recruitment; IRAK1/4 complex; NF-κB translocation; MAPK cascade	Inflammatory mediators: IL-6, TNF-α; Enzymes: COX-2, MMP-9	Enhanced neuronal apoptosis; Glial scar formation	(89–91)
	TNF-R1	TNF-α (early inflammatory response)	TRADD-mediated signaling; TRAF2 recruitment; NF-κB activation; JNK pathway	Cytokines: IL-1β, IL-6; Chemokines: CCL2; Enzymes: iNOS	Oxidative stress enhancement; Neuronal death propagation	(92–94)
Purinergic Receptors	P2X	Mechanical stress-induced; ATP	Calcium influx; NLRP3 inflammasome activation; MAPK signaling	Cytokines: IL-1β, IL-18; Oxidative species: ROS	Calcium overload; Cell death initiation; Inflammatory cascade amplification	(95–97)
	P2Y	ATP/ADP	Calcium mobilization; ERK activation	Inflammatory mediators: COX-2, TNF-α	Vascular response modulation; Inflammation perpetuation	(98, 99)
Chemokine Receptors	CCR2	CCL2 (microglial activation); CCL2 (monocyte infiltration)	PI3K/AKT pathway; MAPK cascade	Chemokines: CCL2; Proteases: MMP-2/9;	Enhanced inflammatory cell recruitment; Tissue damage progression; BBB integrity compromise	(100, 101)
Complement Receptors	C3aR/C5aR	C3a/C5a; Coagulation cascade products	G-protein signaling; PKC activation; MAPK pathway; NF-κB signaling	Cytokines: IL-1β, TNF-α; Chemokines: CCL2/5	Neuroinflammation enhancement; Complement cascade amplification	(102, 103)
Damage Recognition	RAGE	HMGB1; S100β;	NF-κB activation; MAPK signaling; JAK/STAT pathway	Cytokines: TNF-α, IL-1β; Growth factors:VEGF Oxidative species: ROS	Chronic inflammation promotion; Vascular dysfunction	(104–109)
Death Receptors	Fas	FasL (damaged cells); Inflammatory cell expression	FADD recruitment; Caspase-8 activation; NF-κB signaling	Cytokines: TNF-α, IL-1β	Apoptotic pathway activation; Tissue damage enhancement	(110, 111)
Oxidative Stress	NOX4	IL-1β;IFN-γ; NOX; Cellular debris	PKC activation; MAPK cascade; NF-κB pathway	Oxidative species: ROS, NO; Cytokines: TNF-α	Oxidative damage amplification; Mitochondrial dysfunction	(45, 112, 113)

HSP, Heat Shock Protein; PI3K, Phosphatidylinositol 3-Kinase; COX-2, cyclooxygenase-2; CCL2, C-C Motif Chemokine Ligand 2.

#### 3.1.1 IL-1β

Reactive astrocytes predominantly produce pro-inflammatory cytokines, including IL-1β, TNF-α, and interleukin-6 (IL-6). IL-1β functions as a potent mediator of inflammatory responses, inducing the expression of other cytokines and promoting neuronal damage. TBI induces cellular damage, resulting in the release of DAMPs, including ATP, HMGB1, and uric acid crystals (114). DAMPs bind to PRRs on astrocytes, initiating signaling cascades that upregulate pro-IL-1β gene expression and cellular IL-1β production (115). Subsequent to the initial activation signal, a secondary stimulus (e.g., additional DAMPs) triggers the assembly of the NLRP3 inflammasome complex, comprising NLRP3, the adaptor protein ASC, and pro-caspase-1. Within the inflammasome, pro-caspase-1 is activated to caspase-1, which subsequently cleaves pro-IL-1β into its mature, active form (116, 117). This process represents the classical activation pathway. Current research demonstrates that NLRP3 inflammasome activation initiation is complex, involving transcriptional and post-translational mechanisms, and necessitating multiple protein binding partners (118, 119). NLRP3 inflammasome activation induces pore formation in the cell membrane, facilitating the release of mature IL-1β into the extracellular space (120).

Extensive research demonstrates that IL-1β indirectly activates STAT3 as an upstream regulator (121). IL-1β amplifies STAT3 signaling through NF-κB; activated NF-κB translocates to the nucleus and downregulates the expression of suppressor of cytokine signaling 3 (SOCS3), a protein that inhibits STAT3 signal transduction. The decrease in SOCS3 levels enhances STAT3 susceptibility to phosphorylation and activation, promoting its nuclear translocation and transcriptional activity (122). Additionally, IL-1β increases local chromatin accessibility, enhancing STAT3’s impact on interleukin-17a/f (Il17a/f) loci (123). Several studies indicate that the Janus kinase 2 (JAK2)-STAT3 pathway is a crucial mechanism mediating IL-1β release from astrocytes. JAK2 participates in fundamental cellular processes, including apoptosis, autophagy, and proliferation. The JAK2-STAT3 pathway is activated upon pro-inflammatory cytokine binding to their respective membrane receptors (124). Activated JAK2 phosphorylates tyrosine residues on STAT3, inducing STAT3 dimerization (125). The phosphorylated STAT3 dimers subsequently translocate to the nucleus (126, 127). Within the nucleus, STAT3 binds to the IL-1β gene promoter region, initiating its transcription. The resulting IL-1β mRNA is translated into protein and subsequently released into the extracellular space via exocytosis (116, 128). Inhibition of the JAK2-STAT3 pathway significantly reduces IL-1β production by astrocytes in diverse disease models (128, 129).

#### 3.1.2 TNF-α

TNF-α is initially synthesized as a transmembrane protein, termed ‘transmembrane TNF-α (tmTNF-α)’ or ‘pro-TNF-α’. Analogous to IL-1β, NF-κB regulates the upregulation of TNF-α gene expression. Additionally, inflammation activates the MAPK pathway. Activated MAPK translocates to the nucleus, where it phosphorylates transcription factors such as activator protein 1 (AP-1) or NF-κB. These phosphorylated transcription factors subsequently bind to the TNF-α gene promoter, enhancing its transcription and facilitating TNF-α production by astrocytes (130, 131). TNF-α converting enzyme (TACE), a metalloproteinase, specifically recognizes and cleaves the transmembrane structural domain of tmTNF-α at specific sites. This cleavage releases soluble TNF-α (sTNF-α), which is subsequently processed and secreted by astrocytes via the classical secretory pathway (132). The membrane-bound form of TNF-α (tmTNF-α), signaling primarily via TNF receptor 2 (TNFR2), mediates protective and reparative effects. Conversely, the soluble form (sTNF-α), signaling primarily via TNF receptor 1 (TNFR1), promotes pro-inflammatory and detrimental functions. Upon release into the extracellular space, sTNF-α binds to TNFR1 on target cells, triggering a downstream signaling cascade. This cascade includes pathways such as NF-κB, MAPKs (p38 MAPK, c-Jun N-terminal kinase [JNK]), and PI3K (133). In astrocytes, TNF-α binding to TNFR1 upregulates glutaminase expression and increases glutamate production. This interaction also induces glutamate cytotoxicity, enhances the release of extracellular vesicles, inhibits glutamate uptake, and elevates extracellular glutamate levels (134, 135). During excessive neuroinflammation, TNF-α initiates the apoptotic pathway. Binding to TNFR1 leads to recruitment of junction proteins such as TNF receptor-associated death domain protein (TRADD), which activates the apoptotic cascade. This cascade includes the NF-κB pathway and involves cysteine-dependent aspartate-directed proteases (caspases), ultimately leading to neuronal apoptosis (136–138). TNF-α also triggers necroptosis, a caspase-independent form of programmed cell death, by activating receptor-interacting serine/threonine-protein kinase 1 (RIPK1) (139).

TNF receptor 2 (TNFR2) is predominantly expressed in immune and glial cells, mediating the neuroprotective and neurorestorative effects of TNF-α. In the CNS, TNFR2 is predominantly found in neuroglia, with highest expression in microglia (140, 141). TNFR2 expression in astrocytes may promote myelin regeneration through the induction of cytokine expression (142). While TNF-α binding to TNFR1 is associated with pro-apoptotic effects, TNFR2 can also promote cell death pathways, depending on the cellular environment and signaling complexes involved. A recent study demonstrates that reactive astrocytes undergo necroptosis mediated by receptor-interacting protein kinase 3 (RIPK3) and mixed lineage kinase domain-like protein (MLKL) (143). Another study showed that TNFR2 signaling triggers apoptosis in the presence of the adaptor molecule receptor-interacting protein (RIP) (144). These findings suggest that TNF-α binding to TNFR2 may lead to necroptosis of astrocytes under certain conditions.

TNF-α affects intercellular gap junctions through multiple pathways (145). For instance, TNF-α stimulation of spinal cord astrocytes significantly reduces connexin 43 (Cx43) expression and gap junction function through activation of JNK (146). Similarly, a study on corneal endothelial cells reported that TNF-α disrupts gap junctional intercellular communication (GJIC) in the corneal endothelium by interfering with the connection between Cx43 and zonula occludens-1 (ZO-1). In a study of cerebral hemorrhage, TNF-α was shown to activate the NF-κB pathway, promoting the binding of p65 to the promoter region of the aquaporin-4 gene. This enhances aquaporin-4 expression, ultimately reducing astrocyte viability and causing cellular edema (147, 148). In conclusion, the role of TNF-α in neuroinflammation appears to be predominantly detrimental, with damaging effects on neurons, glial cells, and endothelial cells. The most compelling evidence for the role of TNF-α in neuroinflammation and CNS disorders is derived from experimental and clinical studies investigating the therapeutic blockade of this cytokine.

#### 3.1.3 IL-6

The molecular mechanism and signaling pathway of IL-6 production by reactive astrocytes is a complex, multistage process involving the synergistic action of multiple signaling molecules and transcription factors. Similar to the mechanisms of IL-1β and TNF-α production, this process typically initiates with cell surface receptors recognizing specific stimulus signals, such as DAMPs, pathogen-associated molecular patterns (PAMPs), or inflammatory factors (115, 149). These stimuli bind to PRRs, such as IL-1 receptor (IL-1R) or TNF receptor (TNFR), triggering intracellular signaling cascades. IL-6 mediates pro-inflammatory effects in the CNS by initially binding to its specific receptor, IL-6R, forming the IL-6/IL-6R complex. This complex subsequently binds to the signaling protein glycoprotein 130 (gp130), triggering an intracellular signaling cascade. gp130 dimerization activates associated Janus kinases (JAKs), primarily JAK1, JAK2, and tyrosine kinase 2 (TYK2) (150). IL-6 also utilizes gp130 to activate the PI3K-Akt pathway and the Ras-RAF-MEK-extracellular signal-regulated kinase 1/2 (ERK1/2) pathways, further enhancing inflammatory response and cell survival (151, 152). Notably, IL-6 can also act on cells that do not express membrane-bound IL-6R through a trans-signaling mechanism (153). Soluble IL-6R (sIL-6R) binds to IL-6, forming a complex that can bind to widely expressed gp130 and activate signaling, a process known as IL-6 trans-signaling (154, 155). In the neuroinflammatory environment, activated astrocytes and microglia produce substantial amounts of IL-6, which amplifies the inflammatory response through autocrine and paracrine effects (156, 157). IL-6-induced STAT3 activation promotes reactive proliferation of astrocytes and formation of glial scarring (158, 159). Additionally, IL-6/STAT3 signaling upregulates the expression of various cell adhesion molecules (e.g., intercellular adhesion molecule-1 [ICAM-1] and vascular cell adhesion molecule-1 [VCAM-1]) and promotes leukocyte infiltration into the CNS (18). In neurons, IL-6 can increase neuronal excitability by modulating the function and expression of N-methyl-D-aspartate (NMDA) receptors (160, 161). While IL-6 can increase neuronal excitability under certain conditions, its effects are complex and context-dependent, potentially acting as a neuroprotective agent in other situations depending on the concentration and cellular environment (160). IL-6 also induces the expression of COX-2 and increases the production of prostaglandin E2 (PGE2), further amplifying the inflammatory response (162, 163). The IL-6 signaling pathway is subject to strict negative feedback regulation, including the induction of SOCS3 expression and activation of protein tyrosine phosphatases (e.g., Src homology region 2 domain-containing phosphatase-2 [SHP2]) (164, 165). These negative regulatory mechanisms limit the pro-inflammatory effects of IL-6 under normal physiological conditions but may be disrupted in chronic neuroinflammatory states, leading to a sustained inflammatory response (166, 167).

### 3.2 Chemokines

Astrocytes, as the main immune effector cells in the CNS, produce and release a variety of chemokines through complex molecular mechanisms and play a key role in neuroinflammatory responses. Injury stimuli activate PRRs on astrocytes, such as TLRs and RAGE receptors, triggering downstream signaling pathways such as NF-κB and MAPK cascades, inducing the transcription and expression of chemokine genes. These chemokines include, but are not limited to, CCL2, CCL5, CXCL1, and CXCL10. By binding to the corresponding chemokine receptors, they attract peripheral immune cells such as neutrophils, monocytes, and T cells to migrate to the injury site. The production of chemokines can further activate other glial cells in the microenvironment, forming a positive feedback loop and amplifying the inflammatory response. This process may be beneficial in the acute phase to clear cellular debris and repair tissue, but sustained chemokine release may lead to chronic inflammation and exacerbate neuronal damage. In addition, certain chemokines such as CCL2 are also involved in regulating the permeability of the BBB and affecting the degree of infiltration of inflammatory cells. Therefore, precise regulation of chemokines released by astrocytes is of great significance for the prognosis of TBI.

#### 3.2.1 CCL2 (MCP-1)

Chemokines play a crucial role in directing cell migration in various processes, including angiogenesis and inflammatory responses. In the inflammatory milieu, chemokines act synergistically with selectins and integrins to facilitate leukocyte recruitment to inflammatory sites. Within the CNS, multiple cell types are capable of chemokine production. Astrocytes serve as the primary source of CCL2 in experimental models of TBI, endotoxemia, and multiple sclerosis (168). CCL2, also known as monocyte chemoattractant protein-1 (MCP-1), is a chemokine that plays a pivotal role in binding to the CCR2 receptor and facilitating monocyte recruitment to inflammatory sites (169). Astrocytes contribute to the regulation of leukocyte homing to the inflamed CNS. IL-1β and TNF-α stimulate CCL2 production in astrocytes in a time- and concentration-dependent manner (170). IL-1β activates IκB kinase (IKK), and subsequently, the IKK complex activates NF-κB by phosphorylating the IκB molecule, leading to its degradation and the release of NF-κB (171). The liberated NF-κB translocates to the nucleus, where it binds to specific sequences in the regulatory elements of inflammation-related genes, such as CCL2, thereby activating their transcription (47, 172). In contrast to IL-1β, TNF-α primarily induces CCL2 production through activation of the intracellular MAPK signaling pathway (173).

#### 3.2.2 CXCL10

The activation and production process of CXCL10 in astrocytes following TBI parallels that of CCL2, as previously described. Upon production and secretion, CXCL10 exerts multifaceted functions in neuroinflammatory and repair processes following TBI. Primarily, CXCL10 functions as a chemokine, attracting inflammatory cells including monocytes, natural killer (NK) cells, and T helper 1 (Th1) cells from the periphery into the CNS (174). Furthermore, CXCL10 can modulate tight junction proteins, potentially leading to BBB disruption. A study demonstrated that incubation of brain microvascular endothelial cells with CXCL10 significantly reduced the expression of Claudin-5 and Occludin, key components of tight junction proteins (175). Additionally, CXCL10 plays a role in modulating neuronal survival and apoptosis. Studies on spinal cord injury have shown that CXCL10 activates CXCR3 receptors during the acute phase, elevating intracellular calcium levels and triggering the release of cytochrome c from mitochondria. This cascade subsequently activates caspase-9, followed by caspase-3 activation, ultimately resulting in neuronal apoptosis (176) (177).

In-depth exploration of the CXCL10 production mechanism and signaling pathway reveals a highly complex and precisely regulated process. The interplay of multiple signaling pathways forms a complex regulatory network, ensuring rapid and precise CXCL10 expression in response to microenvironmental changes following TBI (178). In addition to the NF-κB pathway, the Janus kinase-signal transducer and activator of transcription (JAK-STAT) signaling cascade plays a pivotal role in CXCL10 production (179). The binding of interferon-γ (IFN-γ) to its receptor activates JAK1 and JAK2, resulting in STAT1 phosphorylation. Subsequently, phosphorylated STAT1 molecules dimerize and translocate to the nucleus, where they bind to the Gamma-Interferon Activation Site (GAS), thereby promoting CXCL10 transcription. Furthermore, the activation of p38 MAPK and extracellular signal-regulated kinases 1/2 (ERK1/2) pathways contributes to the regulation of CXCL10 expression (180). These pathways modulate CXCL10 expression either by influencing transcription factor activity or by directly acting on the CXCL10 gene promoter region.

### 3.3 Anti-inflammatory cytokine

Activated astrocytes modulate neuroinflammatory responses and promote tissue repair through the release of various anti-inflammatory substances. Among these, TGF-β represents a key anti-inflammatory factor secreted by astrocytes. Post-TBI, blood-derived factors such as albumin and fibrinogen stimulate astrocytic TGF-β secretion (181). TGF-β reduces the release of pro-inflammatory cytokines (including IL-6, IL-1β, and TNF-α) and suppresses the pro-inflammatory (M1) microglial phenotype through Smad pathway activation. Additionally, TGF-β enhances the neuroprotective functions of astrocytes via p-Smad2 signaling upregulation, promoting neurite outgrowth and glial scar formation to limit injury spread. While some studies suggest TGF-β may exacerbate fibrosis or inhibit neuroplasticity under certain conditions, its overall anti-inflammatory and reparative roles in TBI are widely acknowledged (182).

IL-10, another crucial anti-inflammatory mediator, is released by activated astrocytes through TLR4 signaling or endotoxin tolerance mechanisms (183). IL-10 effectively reduces pro-inflammatory cytokine secretion from both astrocytes and neighboring microglia by blocking NF-κB and JNK/AP-1 pathways. Furthermore, IL-10 promotes microglial polarization toward the M2 anti-inflammatory phenotype, enhancing phagocytic function and post-injury repair (184). Clinical studies confirm that elevated IL-10 levels strongly correlate with reduced cerebral edema and neuronal death following TBI (184).

miR-873a-5p represents the third key anti-inflammatory mediator, present in astrocyte-derived exosomes and functioning through intercellular communication. This miRNA reduces the release of inflammatory mediators such as IL-6 and IL-1β by directly targeting pro-inflammatory signaling molecules and blocking NF-κB nuclear translocation (185). Additionally, through ERK signaling inhibition, miR-873a-5p induces M2 microglial differentiation, enhancing anti-inflammatory and neuroregenerative functions. Animal studies demonstrate that miR-873a-5p delivery significantly improves post-TBI motor and cognitive deficits (186).

## 4 Astrocyte metabolism in TBI

### 4.1 TBI induces metabolic reprogramming of astrocytes

Following TBI, astrocytes undergo significant metabolic reprogramming. This process begins with massive uptake of neuron-released glutamate through Na^+^-dependent transporters (EAAT1), subsequently activating Na^+^/K^+^-ATPase to maintain ionic gradients, leading to rapid cellular ATP depletion. Additionally, glutamate conversion to glutamine further intensifies energy demands (31). During this process, astrocytes exhibit cancer cell-like metabolic characteristics, known as the Warburg effect: enhanced glycolysis with suppressed OXPHOS, closely associated with mitochondrial dysfunction and ROS accumulation (187).

At the molecular level, βA1-crystallin regulates mitochondrial function by modulating PTP1B activity; its deficiency exacerbates mitochondrial oxidative stress, reduces OXPHOS efficiency, and leads to compensatory glycolysis enhancement (188). Simultaneously, the energy sensor AMPK activates during ATP deficiency, reducing mitochondrial biosynthesis and promoting glycolysis through mTOR pathway inhibition (189). TBI-induced neuroinflammation further intensifies this metabolic reprogramming, with activated microglia releasing pro-inflammatory factors like IL-6 and TNFα, enhancing glycolysis while suppressing OXPHOS through TLR signaling pathways (190). Moreover, BBB disruption-induced microenvironment dysregulation aggravates metabolic imbalance (191).

During metabolic reprogramming, key glycolytic enzymes including HK, PFK, and LDH show significantly increased activity, promoting glucose conversion to lactate (192). Astrocytes release excess lactate through MCT4; although lactate can serve as an energy substrate for neurons, its excessive accumulation leads to extracellular acidification and neuronal dysfunction (189). Simultaneously, TBI directly impairs mitochondria, causing decreased membrane potential and reduced ETC complex activity (187). Impaired OXPHOS not only reduces ATP production but also releases ROS that further damage mitochondria, creating a vicious cycle (193). Additionally, glycolytic intermediates (such as citrate) may indirectly suppress OXPHOS by inhibiting PDH, reducing acetyl-CoA entry into the TCA cycle (194).

### 4.2 Role of astrocyte lactate metabolism in neuroprotection

Following TBI, metabolic coupling between astrocytes and neurons plays a crucial role in neural repair. When neuronal mitochondrial dysfunction leads to decreased OXPHOS efficiency, astrocyte-derived lactate can enter neuronal mitochondria via MCT2, converting to pyruvate for participation in the TCA cycle, thereby generating ATP to maintain neuronal membrane potential and synaptic activity (195). Particularly under ischemic conditions, astrocytic glycogen reserves effectively delay axonal function loss, while exogenous lactate supplementation significantly reduces brain atrophy and improves long-term behavioral outcomes (196).

Astrocyte-derived lactate exhibits multiple neuroprotective effects. First, lactate promotes glutathione regeneration by elevating neuronal NAD+ levels, thus alleviating oxidative stress-induced neuronal damage (197). Second, lactate participates in ionic gradient regulation, effectively reducing post-TBI cellular edema through its metabolically generated osmotic balancing effects (198).In terms of synaptic plasticity and cognitive function recovery, ANLS plays an indispensable role. In the hippocampal region, ANLS activation is essential for inducing LTP, where lactate promotes synaptic remodeling by modulating NMDAR activity and CREB signaling pathways (199). Post-TBI, increased hippocampal glycogenolysis leads to elevated lactate release, which positively correlates with the expression of memory-related genes (such as Arc and c-Fos). Studies have shown that ANLS blockade significantly impacts cognitive function recovery (200).

Therapeutic strategies targeting lactate metabolism show promise in neuronal recovery. Hypertonic sodium lactate administration improves CBF and reduces cerebral edema while enhancing cognitive recovery (201). Combining pyruvate with PDK inhibitors like DCA optimizes lactate production for neuronal energetics (202). Modulation of lactate transporters - promoting MCT4-mediated export and MCT1-mediated neuronal uptake - improves metabolic coupling (203). BCATm inhibition reduces astrocytic BCKA accumulation, mitigating glutamate toxicity (204).Alternative energy substrates, including β-HB and succinate, support neuronal metabolism by directly entering the TCA cycle and enhancing mitochondrial function (205) (206). PPARγ agonists demonstrate dual benefits by suppressing NF-κB-mediated inflammation and upregulating lactate synthesis. The triterpene Lupeol attenuates oxidative stress through ROS suppression (207).Molecular interventions including MAGL deletion enhance astrocytic 2-AG signaling, promoting anti-inflammatory effects via CB1R and PPARγ pathways (208). Nrf2 activation increases antioxidant capacity through SOD and GSH upregulation, protecting against lactate-induced oxidative damage (182).

### 4.3 Key regulators in astrocyte metabolic changes after TBI

Following TBI, astrocytic metabolic regulation involves multiple signaling pathways. AMPK, a cellular energy sensor, plays crucial regulatory roles post-TBI. During early glucose metabolic depression and subsequent transient enhancement, AMPK phosphorylates GAPDH and PDH to inhibit glycolysis and fatty acid synthesis while promoting mitochondrial oxidative metabolism to alleviate energy crisis (209). In glutamate-toxic environments, AMPK regulates glutaminase activity to maintain the glutamate-glutamine cycle, providing metabolic support for neurons (210). Additionally, AMPK suppresses inflammatory factor release via JAK2/STAT3/NF-κB pathway inhibition and protects astrocytes through MSC-derived exosomes (211). Through resveratrol-mediated AMPK/mTOR axis, AMPK activates autophagy to clear damaged mitochondria and protein aggregates (212). In the metabolic network, AMPK and mTOR form a negative feedback loop through TSC2 phosphorylation (213). Under hypoxic conditions, AMPK cooperates with HIF-1α to enhance glycolytic capacity. The mTOR complexes (mTORC1/2) exhibit temporal-spatial dependent effects post-TBI. Early mTORC1 hyperactivation exacerbates glial scarring (214), while during recovery, it supports astrocyte repair through lipid synthesis (215). mTORC2 promotes cell survival via Akt signaling (216). HIF-1α drives astrocytic metabolic reprogramming by upregulating GLUT1 and LDHA, enhancing glycolysis to produce lactate as an alternative neuronal energy substrate (210). While HIF-1α promotes angiogenesis through VEGF, its sustained activation may worsen cerebral edema (216). It also reduces pro-inflammatory factors via NF-κB pathway inhibition (214) and activates antioxidant enzymes like SOD2 and HO-1 (217).

Therapeutic strategies targeting AMPK show promise. AICAR effectively activates AMPK (218), while metformin indirectly activates it through mitochondrial complex I inhibition (219). Curcumin reduces LPS-induced astrocytic inflammation via AMPK activation (220). Metabolic substrate regulation, including pyruvate supplementation (221) and BCAA intervention (204), shows efficacy. Combined approaches demonstrate advantages: PGC-1α agonists synergize with AMPK to enhance mitochondrial biogenesis (222), while mTORC1 inhibition augments AMPK-mediated mitochondrial quality control (223). Intervention timing is crucial: acute phase (≤6h post-TBI) should focus on glucose oxidation restoration and AMPK activation, while subacute phase (3-7d) emphasizes mitochondrial autophagy and antioxidant defense (221).

## 5 Therapeutic studies targeting pro-inflammatory astrocytes after TBI

### 5.1 Limitations and progress of research methods on TBI mechanisms

Real-time *in vivo* monitoring of astrocytic activity faces multiple technical challenges. Regarding spatiotemporal resolution, while diffusion MRI can detect functional changes in astrocytes, its resolution is insufficient to capture subcellular dynamic changes. Two-photon microscopy provides higher resolution but is limited by optical penetration depth and tissue scattering in live experiments (224). Similarly, calcium imaging can record astrocytic Ca²^+^ signals but may miss rapid transient activities (225), while photoacoustic and bioluminescence imaging’s low sampling rates might overlook millisecond-scale signal fluctuations (226).At the experimental operation level, invasive devices such as fiber recording and two-photon imaging require head fixation or probe implantation, which not only restricts natural mouse behavior but may also activate local astrocytes (122, 125). Some imaging techniques require anesthesia, which suppresses neuronal and astrocytic activity, affecting the observation of post-TBI pathological processes (225, 226).Cellular heterogeneity and signal specificity constitute another significant challenge. Astrocytes in different brain regions exhibit marked differences in calcium signal patterns, morphology, and function, making it difficult for current technologies to distinguish these subtypes’ specific responses in TBI (227, 228). Furthermore, calcium indicators may be affected by pH fluctuations and struggle to differentiate between overlapping neuronal and astrocytic signals (225, 229). Regarding technical integration and data analysis, differences in sampling rates and spatial coverage among multimodal data make temporal alignment challenging (226), while high-resolution imaging generates massive datasets requiring sophisticated algorithms (230). In physiological and pathological state monitoring, variations in TBI model injury characteristics affect the uniformity of astrocytic responses (231), and current technologies struggle to achieve long-term dynamic tracking of chronic astrocyte activation (232).

Multi-omics approaches play a crucial role in studying astrocyte subtypes following TBI. scRNA-seq represents a crucial breakthrough in TBI research. A 2018 study published in Nature Communications utilized single-cell analysis to reveal early activation of microglia and astrocytes following concussion, elucidating their driving role in neuroinflammatory cascade reactions (233). This technology also successfully identified susceptibility differences among specific neuronal subpopulations in axonal injury (234). Spatial transcriptomics, integrating LCM and *in situ* sequencing, enables researchers to precisely localize molecular characteristics of cells surrounding injury sites, particularly advancing understanding of spatial correlations between endothelial cells and pericytes signaling pathways in BBB disruption regions post-TBI (235). Proteomics and metabolomics analyses have identified multiple subtype-specific markers. Following TBI, upregulated AQP4 expression leads to edema, while decreased GLT-1 expression induces glutamate toxicity (11, 182, 236). Notably, astrocyte-derived exosomes carrying molecules such as miR-148a-3p influence neuroinflammation by modulating microglial phenotypes (186). Epigenomic studies have revealed that Nrf2 deficiency promotes astrocyte transformation toward pro-inflammatory phenotypes by enhancing NF-κB signaling (182). Bioinformatics analysis plays a crucial role in target screening. Differential expression analysis identifies subtype-specific genes, while KEGG/GO enrichment analysis clarifies functional pathways (237, 238). Protein interaction network construction has identified hub genes such as NF-κB and STAT3 (238). Machine learning models have revealed PPAR-γ as a core regulatory factor for astrocyte protective functions (208, 239). Currently validated therapeutic targets include: AQP4 inhibitors for reducing brain edema (11, 236), GLT-1 agonists for restoring glutamate homeostasis (182), 2-AG metabolic regulation through MAGL inhibition or CB1 receptor activation for enhanced neuroprotection (208), and PPAR-γ agonists for modulating inflammatory responses and improving cognitive function (118).

The application of optogenetics has further expanded the depth of TBI research. Through selective activation or inhibition of specific neuronal populations using light-sensitive proteins (such as ChR2 or NpHR), researchers have successfully decoded post-TBI neural network reorganization mechanisms and discovered that modulating PFC-hippocampal circuits effectively improves post-traumatic memory deficits (240). A 2024 Brain Circulation review highlighted that combining optogenetics with closed-loop feedback systems enables real-time regulation of post-traumatic epilepsy-associated aberrant neural oscillations (241).2P microscopy technology, particularly miniaturized fiber-based systems, enables researchers to observe calcium signaling dynamics in freely moving mice, capturing millisecond-scale neuronal activity changes post-TBI. This technology has not only revealed the spatiotemporal propagation patterns of post-injury CSD and its association with secondary injury but also achieved simultaneous “manipulation-recording” observations through integration with optogenetic stimulation, thoroughly elucidating the mechanistic roles of specific neural circuits in injury repair processes (240).

### 5.2 Clinical relevance of astrocyte activation and TBI

Clinical manifestations following TBI are closely associated with astrocyte activation. In epilepsy, particularly PTE, multiple mechanisms are involved: dysfunction of astrocytic potassium channel Kir4.1 leads to extracellular K+ accumulation, resulting in elevated neuronal excitability (182, 242, 243); simultaneously, reduced GLT-1 expression causes glutamate homeostasis disruption, leading to synaptic glutamate accumulation and NMDA receptor activation, triggering excitotoxicity (182). In diffuse TBI models, the expansion of atypical astrocytic regions positively correlates with seizure frequency (15). Regarding cognitive and motor dysfunction, sustained release of IL-6 and TNF-α suppresses synaptic plasticity, resulting in hippocampus-dependent memory impairment (244). Additionally, inflammation-activated astrocytes exhibit abnormal mitochondrial fission, causing energy metabolism disorders and affecting neuronal survival (245). In the pathological process of cerebral edema and elevated ICP, altered polarity of AQP4 in reactive astrocytes exacerbates vasogenic edema (246).

Astrocytes exert dual effects on TBI clinical outcomes. In the acute phase of moderate TBI, astrocytes provide protection through secretion of various neuroimmune modulators, not only limiting inflammatory spread but also maintaining ionic homeostasis and reducing cortical tissue loss (247, 248). However, in the chronic phase, persistently activated astrocytes release pro-inflammatory mediators, leading to secondary neuronal degeneration associated with long-term cognitive deficits and cerebral atrophy (249). Regarding epileptogenesis risk, atypical astrocytic responses following diffuse TBI closely correlate with epilepsy latency period, with approximately 30% of mice developing spontaneous seizures weeks post-injury. Clinical studies indicate GLT-1 expression levels may serve as biomarkers for PTE prediction (15). For functional recovery and prognosis, inflammatory mediator levels negatively correlate with outcomes, with elevated IL-1β and TNF-α concentrations associated with reduced GOSE scores, indicating poor neurological recovery (250, 251).

Astrocyte-related biomarkers play crucial roles in the diagnosis and prognostic evaluation of TBI. GFAP, as a key marker, reaches peak blood levels around 24 hours post-injury, closely correlating with injury severity (252). The TRACK-TBI study demonstrated that serum GFAP exhibits superior sensitivity (AUC 0.88) for detecting intracranial lesions compared to UCH-L1 (AUC 0.71). In acute diagnosis, GFAP shows excellent diagnostic performance (AUC 0.91), particularly in patients with CT-visible lesions (253). While GFAP alone has limited ability to predict 3-month outcomes (AUC 0.65-0.74), its combination with UCH-L1 significantly enhances predictive value (AUC 0.94) (254). Sustained GFAP elevation may indicate secondary injury or chronic neuroinflammation, correlating with neurodegenerative changes in chronic traumatic encephalopathy (255). However, GFAP application has certain limitations. Healthy astrocytes show regional variations in GFAP expression, with high expression in the hippocampus and low expression in the thalamus, and its expression is dynamically regulated by environmental factors such as mechanical stretch (256, 257). Additionally, GFAP release may occur in extra-CNS injuries, necessitating differential diagnosis through imaging or neurological symptoms (255).

Another important marker, S100β, primarily secreted by astrocytes and oligodendrocytes, participates in Ca²^+^ signaling regulation and neuroprotection, though excessive release may lead to neurotoxicity (258). S100β exhibits unique release kinetics, reaching plasma peaks within 1–3 hours post-injury with a half-life of approximately 30 minutes, making it suitable for early detection (259). Although S100β shows high sensitivity in mTBI diagnosis, its specificity is limited by peripheral sources (252). S100β levels correlate with cerebral edema severity and BBB disruption, but its independent ability to predict long-term outcomes remains limited (260). Notably, S100β may elevate in non-neurological conditions, necessitating combination with other markers for improved diagnostic accuracy (252).

### 5.3 Advances in astrocyte-targeted therapy

#### 5.3.1 Treatment mechanisms

A1 astrocytes are critical therapeutic targets in neurological disorders. While BBB presents a significant delivery challenge, several strategies have emerged to overcome this barrier. Small molecules with optimal lipophilicity can directly penetrate the BBB. The HDAC6 inhibitor LASSBio-1911 promotes A1-to-A2 phenotype conversion and reduces inflammatory mediator release (261). Similarly, FLX acts through astrocytic 5-HT2B/β-arrestin2 signaling to suppress A1 activation (262). Receptor-mediated targeting has shown promise, with GLP-1R agonist semaglutide achieving CNS delivery while maintaining BBB integrity (263). Advanced delivery systems including NPs and EVs enhance therapeutic efficiency, particularly EV-delivered HDAC inhibitors which show improved astrocytic accumulation with reduced systemic effects (261). Liposomal anti-inflammatory agents, especially anti-C3 antibodies, demonstrate enhanced delivery through nano-carrier conjugation (25).

Off-target considerations are crucial in therapeutic development. While selective HDAC6 inhibitors like Tubastatin A show specificity, their BBB penetration may affect multiple CNS substrates. Altered microtubule acetylation can impact both target and non-target cells, potentially affecting astrocyte migration and synaptic plasticity (264). Long-term FLX administration may modulate neuronal excitability through astrocytic ATP release and Kir4.1 channel inhibition, risking edema or seizure threshold alterations (265). GLP-1R agonists, due to widespread receptor distribution in hypothalamic and amygdalar regions, may influence reward circuitry through dopaminergic modulation. Their acute versus chronic effects on stress responses and BDNF expression require individualized clinical assessment (266).

Recent studies reveal multiple mechanistic pathways in targeting astrocytic NLRP3 inflammasome. D2R activation inhibits NLRP3 inflammasome by enhancing β-arrestin2-NLRP3 interaction (55). Studies under hyperoxic conditions have elucidated NLRP3 inflammasome-mediated pyroptosis pathways in astrocytes, providing therapeutic strategy insights (267). ASCT2 plays a crucial role in glial inflammatory responses, with its inhibitor talniflumate revealing novel regulatory mechanisms through ASCT2-NLRP3 binding disruption (268). These findings offer new therapeutic targets for neuroinflammation, particularly in modulating astrocytic inflammatory and secretory pathways (269).Caspase-1 inhibitors demonstrate significant therapeutic potential in inflammasome regulation. These compounds specifically target glial inflammasome activation, effectively reducing pro-inflammatory cytokine production (270). Their high specificity and affinity modulate cytokine release following astrocytic activation (271). Through Nlrp1 inflammasome regulation, they effectively control cognitive dysfunction and inflammatory responses (272). Specifically, YVAD treatment significantly reduces pro-inflammatory mediators while providing neuroprotection (273).

STAT3 inhibitors demonstrate significant therapeutic potential in neurological disorders. Stattic, targeting STAT3’s SH2 domain, inhibits phosphorylation and dimerization (274). In AD models, it suppresses astrocytic STAT3 activation, reducing Aβ-induced neuronal death (275). In SCI models, Stattic decreases astrocytic chemokine expression (CX3CL1, CXCL10) in dorsal horn, alleviating mechanical pain (274). For PD models, it inhibits H_2_O_2_-induced GFAP expression and pro-inflammatory cytokine release (276).H-4-54 (K_D_=300nM) effectively inhibits STAT3 phosphorylation and downstream transcription (142). In APP/PS1 mice, it reduces pSTAT3-positive astrocytes near plaques and enhances microglial morphological complexity, though amyloid clearance effects remain limited (277).AG490, a JAK2 inhibitor, reduces STAT3 phosphorylation. In AD models, it decreases astrocyte activation through JAK2/STAT3 pathway inhibition, requiring careful dose control to preserve neuronal viability (275). In PD models, it effectively suppresses H_2_O_2_-induced astrocyte activation through STAT1/3 inhibition (276). Indirect STAT3 modulators show promise. ADORA2A agonists like CGS21680 inhibit STAT3/YKL-40 signaling and reduce inflammatory cytokine release. In CCH models, they attenuate astrocyte activation and improve cognition, showing synergy with STAT3 inhibitors (278). Similarly, si-sEH reduces astrocytic inflammatory responses in LPS-induced models through STAT3 modulation (279).

NF-κB, a key transcription factor in astrocytic inflammatory responses, is central to neuroinflammation. Pro-inflammatory factors like TNF-α and IL-1β upregulate inflammation-related genes through NF-κB pathway activation (280). In SCI models, NF-κB activation correlates with astrocytic inflammation, while its inhibition reduces white matter damage and improves recovery (281). NF-κB suppression decreases TNF-α and IL-1β production, with potential cross-regulation through MAPK pathways (282). IL-6 regulation exhibits complex mechanisms. In SCI models, NF-κB inhibition paradoxically increases IL-6 expression, possibly through negative feedback or compensatory STAT3 activation (281). Conversely, in AD models, NF-κB inhibition reduces IL-6 secretion (283). For COX-2 and iNOS regulation, PD168393 reduces their secretion via EGFR phosphorylation inhibition (284). Mn-induced NF-κB activation through NOS2 expression confirms its role in oxidative stress (285).While direct evidence for IL-12, IL-18, and CCL2 regulation is limited, NF-κB likely influences their expression indirectly, with CCL2 upregulation closely linked to NF-κB activation (286). VEGF-B reduces inflammatory factors through NF-κB pathway inhibition (282). Additionally, Nrf2 activators show neuroprotective potential by antagonizing NF-κB’s pro-inflammatory effects (283).

#### 5.3.2 Treatment strategies

Therapeutic strategies for astrocyte targeting have evolved significantly. ADCs and nanocarriers targeting astrocytic surface markers enhance local drug concentrations (287). Novel approaches focus on dual inhibitors targeting astrocyte-neural cell interactions (288, 289), while disease progression stages necessitate specific strategies based on astrocytic inflammasome activation mechanisms (290). NPs demonstrate unique advantages in astrocyte-targeted therapy. For BBB penetration, NPs utilize RMT through TfR or LDLR surface modifications, while FUS with microbubble oscillation enhances penetration efficiency (291). Surface modifications improve targeting specificity; 30nm glucose-coated AuNPs show doubled astrocyte uptake compared to GSH-coated variants, and CSF-derived protein corona increases PLGA NP uptake seven-fold (292). CeNPs effectively reduce A1 astrocyte inflammation and promote myelination via ROS-NF-κB pathway inhibition (26). Compared to traditional treatments like fluoxetine (293), nano-delivery systems offer superior efficacy. MSNs provide pH-responsive drug release with reduced systemic toxicity (294), while PEG-modified Fe3O4 MNPs combine targeted delivery with MRI monitoring capabilities (295).Safety concerns persist, as Ag-NPs may disrupt BBB TJ proteins and impair astrocytic mitochondrial function (296). Ongoing research incorporates scRNA-seq to identify astrocyte subtype markers for developing more selective nanocarriers (297). The CRISPR/Cas9 system, derived from bacterial adaptive immunity, enables precise gene editing through sgRNA-guided targeting (298). In astrocyte research, this technology facilitates gene KO, KI, and epigenetic modulation. Cre-loxP-mediated STAT3 KO reduced glial scarring post-SCI, despite increased inflammation (299), while Smad3 or TGFBR gene editing improved astrocyte function in TBI models (181).CRISPR-mediated KO of TREK-1 or TWIK-1 reduced K+ leak currents in astrocytes (300), and APP KO enhanced Aβ clearance in NDDs (301). HB-EGF KO revealed its protective role under inflammatory conditions (302), while IFN-γ pathway editing affected astrocytic APC capacity (303).

#### 5.3.3 Progress in drugs targeting A1 astrocytes

The transformation of astrocytes into the neurotoxic A1 phenotype following TBI represents a critical factor exacerbating secondary injury. Recent years have witnessed significant progress in targeted therapeutic studies against A1 astrocytes, offering new prospects for improving TBI prognosis. Current research primarily focuses on three directions: modulation of cell signaling pathways, inhibition of inflammatory responses, and promotion of cytoprotective mechanisms. Strategies targeting cell signaling pathways are particularly promising, exemplified by the application of the mammalian target of rapamycin (mTOR) inhibitor, rapamycin. Rapamycin mitigates NLRP3 inflammasome activation by inhibiting the mTOR complex 1 (mTORC1), which directly reduces A1 astrocyte formation and enhances autophagy-mediated removal of damaged cellular components, thereby attenuating neuroinflammation (304–306). This dual mechanism of action confers unique neuroprotective advantages to rapamycin following TBI. Concurrently, significant advancements have been made in the investigation of fingolimod, a sphingosine-1-phosphate (S1P) receptor modulator. Fingolimod primarily influences astrocyte polarization by modulating S1P receptor 1 (S1P1) activity. This modulation not only inhibits the transformation to the A1 phenotype but also promotes the formation of the A2 phenotype, which is conducive to neural repair (307, 308). The regulation of this phenotypic conversion offers novel approaches for optimizing the neural microenvironment following TBI, potentially mitigating inflammation while promoting neuroregeneration.

Regarding the inhibition of inflammatory responses, cytokine therapy and the development of specific inhibitors have emerged as focal points of research. A particularly noteworthy direction is the application of α7 nicotinic acetylcholine receptor (α7nAChR) agonists. Compounds such as GTS-21 can inhibit the NF-κB pathway and STAT3 phosphorylation through α7nAChR activation, thereby attenuating A1 astrocyte activation (309). Furthermore, α7nAChR activation promotes the release of anti-inflammatory factors, such as interleukin-10 (IL-10), establishing a positive feedback loop that augments its anti-inflammatory effects (310, 311). This multifaceted regulatory mechanism endows α7nAChR agonists with significant neuroprotective potential following TBI.

**Figure 2:**
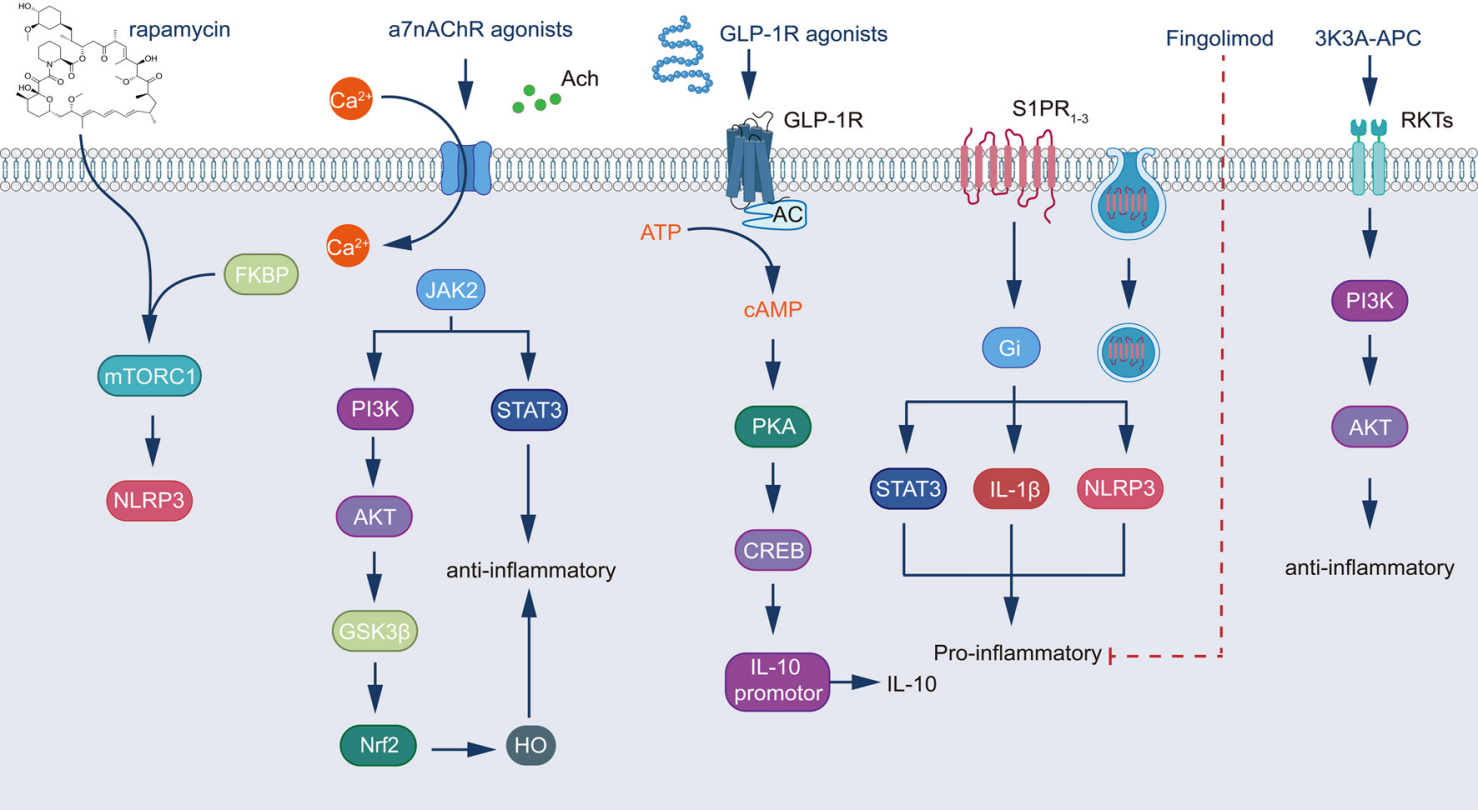
Integration of multiple signaling pathways in the regulation of cellular inflammatory responses. The diagram illustrates five major signaling cascades and their interconnections: (1) The rapamycin pathway, where rapamycin complexes with FKBP to inhibit mTORC1, subsequently modulating NLRP3 inflammasome activity; (2) α7nAChR signaling, initiated by agonist binding and calcium influx, which activates dual pathways - the JAK2/STAT3 cascade and the PI3K/AKT/GSK3β/Nrf2 pathway, culminating in HO expression and anti-inflammatory effects; (3) GLP-1R signaling, triggered by receptor agonist binding, which stimulates adenylate cyclase to produce cAMP, activating the PKA/CREB pathway and inducing IL-10 expression through promoter activation; (4) The fingolimod pathway, operating through S1PR1 and Gi protein activation to regulate STAT3 and the NLRP3/IL-1β pro-inflammatory axis; and (5) 3K3A-APC signaling, which functions through PI3K/AKT activation to promote anti-inflammatory responses. These pathways demonstrate the intricate molecular mechanisms controlling inflammation, featuring key regulatory elements including transcription factors (STAT3, Nrf2, CREB), kinases (PI3K, AKT, JAK2, PKA), second messengers (Ca2+, cAMP), and inflammatory mediators (NLRP3, IL-1β, IL-10). The integration of these signaling cascades reveals multiple therapeutic targets and their respective pharmacological interventions in the modulation of inflammatory responses, highlighting the complex interplay between pro- and anti-inflammatory mechanisms in cellular regulation.

The promotion of cytoprotective mechanisms represents the third crucial research direction, with glucagon-like peptide-1 (GLP-1) receptor agonists garnering particular interest. Recent studies have demonstrated that GLP-1 receptor agonists mitigate inflammation and BBB disruption in experimental stroke models. For instance, the GLP-1 receptor agonist exendin-4 (Ex-4) attenuates astrocytic production of inflammatory factors, including vascular endothelial growth factor A (VEGF-A), matrix metalloproteinase-9 (MMP-9), CCL2, and C-X-C motif chemokine ligand 1 (CXCL-1) (312). Liraglutide, originally developed for diabetes treatment, has been found to play a significant role in neuroprotection (313, 314). In a murine model of ischemic stroke, the GLP-1 agonist semaglutide demonstrated alleviation of BBB disruption by inhibiting C3d+/GFAP+ A1 astrocyte transformation (263). Another noteworthy direction is the application of 3K3A-APC, a variant of activated protein C. 3K3A-APC activates protease-activated receptor 1 (PAR1), and APC signaling through PAR1 differs significantly from thrombin-induced signaling; thrombin effects are mediated by G proteins, whereas APC-induced signaling is β-arrestin-mediated. Activation of PAR1 by 3K3A-APC reduces A1 astrocyte formation and promotes A2 astrocyte transformation. Furthermore, it enhances cell survival by activating the protein kinase B (Akt) signaling pathway and mitigates inflammatory factor production by inhibiting the NF-κB signaling pathway (315–317). This dual anti-inflammatory and neuroprotective property positions 3K3A-APC as a promising drug candidate in TBI treatment research (**Figure 2** Integration of multiple signaling pathways in the regulation of cellular inflammatory responses.).

Recent years have witnessed significant progress in targeted therapies for astrocytes. NLY01, a GLP-1R agonist, effectively blocks A1 astrocyte activation by inhibiting microglial release of IL-1α, TNFα, and C1q. This drug has completed Phase I safety trials for PD treatment and is currently undergoing Phase II trials (NCT04154072) to evaluate its efficacy in early untreated PD patients (318). In AD research, preclinical studies indicate that NLY01 reduces Aβ deposition and astrocyte activation, demonstrating potential in delaying neurodegenerative disease progression through indirect targeting of upstream A1 activation signals. Simvastatin inhibits A1 astrocyte polarization by suppressing HMG-CoA reductase and modulating NR4A2 activity, thereby reducing IL-6 and TNFα production. A Phase II clinical trial (NCT02787590) for moderate PD patients has been completed, with results pending publication (319). Current research focuses on validating the drug’s specific regulatory effects on astrocyte phenotypes. Neflamapimod, a selective p38 MAPK α subtype inhibitor, effectively reduces inflammatory factor release from astrocytes and microglia. In AD treatment, Phase IIa trials have confirmed its ability to lower CSF tau protein levels, and Phase IIb trials (NCT03402659) are ongoing (320, 321). While not directly targeting A1 astrocytes, it may indirectly suppress their activity through neuroinflammation regulation. Regarding combination therapy strategies, the joint application of ibuprofen and Cromolyn demonstrates unique advantages. Ibuprofen inhibits COX-2-mediated inflammatory responses, while Cromolyn blocks microglial activation, collectively reducing A1 astrocyte polarization. This combination therapy is currently undergoing Phase I clinical trials (NCT04570644) for early AD, focusing on safety assessment and biomarker effects (322).

In TBI treatment research, regulatory strategies targeting astrocytes primarily encompass three directions: small molecule drugs, gene therapy, and stem cell transplantation. In the small molecule drug field, TGF-β signaling pathway regulation has shown significant value. Studies reveal that TGF-β exhibits dual effects in TBI: moderate activation suppresses inflammatory responses, while excessive activation leads to glial scar formation and neurite growth inhibition. Fibrinogen activates the astrocytic p-Smad2 pathway by releasing latent TGF-β, and inhibiting this pathway significantly improves neuroplasticity (181). In preclinical studies, TGF-β inhibitor SB431542 has been used to reduce astrocyte proliferation, though its application requires precise control of dosage and administration timing (323). Endothelin receptor antagonist studies demonstrate that post-TBI ET-1 promotes astrocytic STAT3 pathway activation through ETB-R, inducing reactive proliferation and vasoconstriction. ETB antagonist BQ788 has shown effectiveness in reducing cerebral edema and restoring BBB function in animal experiments (324), while decreasing the release of chemokines CCL2 and CXCL1, effectively suppressing neuroinflammation (325). Estrogenic compounds, particularly E2 and its analogs, enhance antioxidant and anti-inflammatory capabilities by activating astrocytic ERs. Notably, phytoestrogen isoflavones significantly reduce post-TBI glial scarring and promote synaptic remodeling (326). Regarding anti-inflammatory and antiepileptic drugs, LEV prevents post-TBI epilepsy by maintaining astrocytic connexin expression (327), while minocycline indirectly modulates inflammation by inhibiting microglial p38 pathway (328).

Breakthroughs in gene therapy primarily focus on CRISPR/Cas9 technology applications. Research indicates that APOE4 allele correction can improve cholesterol metabolism and Aβ clearance dysfunction in APOE4-carrying astrocytes, validated in AD models and potentially applicable to post-TBI neurodegeneration treatment (329). Viral vector-mediated gene delivery, particularly AAV-delivered GDNF/BDNF genes to astrocytes, shows potential in improving dopaminergic neuron function in PD models (330). In stem cell therapy, iPSC-derived astrocyte transplantation demonstrates unique advantages. Using the LRRK2-mutant PD model as an example, transplantation of genetically corrected iPSC-astrocytes effectively reduces α-synuclein aggregation. While early clinical trials have confirmed stem cell transplantation safety, further optimization of cell types and transplantation strategies is needed for functional recovery (329).

## 6 Conclusions and future directions

Exploration of astrocyte activation in the context of TBI has revealed a complex interplay of cellular and molecular mechanisms that significantly influence outcome after injury. This review systematically investigates the pathways triggering astrocyte activation after TBI, the complex mechanisms controlling the release of inflammatory mediators from activated astrocytes, and the multifaceted roles that these inflammatory mediators play in the progression of secondary injury. In addition, we highlight recent advances in targeted therapeutic approaches aimed at modulating pro-inflammatory astrocytes. Activation of astrocytic inflammatory mediators after TBI represents a pivotal moment in the pathophysiology of secondary brain injury. The dual nature of astrocyte reactivity (neuroprotective and neurotoxic) emphasizes the complexity of targeting these cells in therapeutic interventions.

The current state of research presents both promising opportunities and significant challenges. Despite the exponential growth in our understanding of astrocyte biology in TBI, translating this knowledge into effective clinical interventions remains an ongoing endeavor. The heterogeneity of astrocyte responses to injury, coupled with the dynamic nature of inflammation following TBI, requires a nuanced therapeutic approach. Recent advances in understanding the molecular basis of astrocyte inflammasomes activation have paved the way for new therapeutic strategies. Targeted approaches aimed at modulating specific components of the inflammasomes machinery, such as NLRP3 inhibitors and caspase-1 blockers, have shown promise in preclinical models. However, translating these findings into clinically viable therapeutics remains a major challenge. Looking forward, several key areas emerge as priorities for ongoing research: a) A more comprehensive understanding of astrocyte subpopulations and their differential responses to TBI may lead to more targeted and effective therapies. b) Developing methods to selectively modulate pro-inflammatory astrocytes while preserving the beneficial functions of other astrocyte subpopulations. c) Exploring the synergistic effects of simultaneously targeting multiple aspects of the astrocyte inflammasomes pathway in a synergistic manner. d) Drug delivery methods that allow for precise, localized delivery of anti-inflammatory drugs to overcome the BBB and minimize systemic side effects. e) Bridging the gap between preclinical success and clinical application through improved animal models and innovative clinical trial design.

The path forward for TBI research and treatment is undoubtedly challenging, but full of potential. As we continue to unravel the complexities of astrocyte inflammatory mediator activation production, we are getting closer to discovering targeted and effective therapies that can significantly improve the prognosis of patients with TBI. The integration of cutting-edge technologies such as single-cell genomics and advanced imaging with traditional research methods promises to accelerate progress in this critical area. As our understanding of astrocyte biology in TBI continues to evolve, so does the potential for developing targeted therapies. Significant progress in this area is expected in the future, with the potential to further fill the therapeutic landscape for patients with TBI. However, the complex role of astrocytes in maintaining brain homeostasis must be carefully considered to avoid unintended consequences of therapeutic interventions.
